# Tetrahydrobiopterin in Myalgic Encephalomyelitis/Chronic Fatigue Syndrome: A Friend or Foe?

**DOI:** 10.3390/biom15010102

**Published:** 2025-01-10

**Authors:** A. F. M. Towheedur Rahman, Anna Benko, Sarojini Bulbule, Carl Gunnar Gottschalk, Leggy A. Arnold, Avik Roy

**Affiliations:** 1 Milwaukee Institute for Drug Discovery, University of Wisconsin-Milwaukee, 2000 E Kenwood Blvd, Milwaukee, WI 53211, USA; rahman25@uwm.edu (A.F.M.T.R.); abenko@uwm.edu (A.B.); arnold2@uwm.edu (L.A.A.); 2 Research and Development Laboratory, Chemistry Building, 2000 E Kenwood Blvd, Suite #320, Milwaukee, WI 53211, USA; sb@simmaron.com (S.B.); ggottschalk@simmaron.com (C.G.G.); 3 Simmaron Research Institute, 948 Incline Way, Incline Village, NV 89451, USA

**Keywords:** tetrahydrobiopterin (BH4), dihydrobiopterin (BH2), pentose phosphate pathway (PPP), ME/CFS, orthostatic intolerance (OI), oxidative stress, autophagy

## Abstract

Myalgic Encephalomyelitis or Chronic Fatigue Syndrome (ME/CFS) is a chronic multisystem disease characterized by severe muscle fatigue, pain, dizziness, and brain fog. The two most common symptoms are post-exertional malaise (PEM) and orthostatic intolerance (OI). ME/CFS patients with OI (ME+OI) suffer from dizziness or faintness due to a sudden drop in blood pressure while maintaining an upright posture. Clinical research has demonstrated that patients with OI display severe cardiovascular abnormalities resulting in reduced effective blood flow in the cerebral blood vessels. However, despite intense investigation, it is not known why the effective cerebral blood flow is reduced in OI patients. Based on our recent findings, we observed that tetrahydrobiopterin (BH4) metabolism was highly dysregulated in ME+OI patients. In the current review article, we attempted to summarize our recent findings on BH4 metabolism to shed light on the molecular mechanisms of OI.

## 1. Introduction

The coenzyme (5,6,7,8)-tetrahydrobiopterin, commonly known as tetrahydrobiopterin (BH4), is an important cofactor that regulates the biological actions of cellular enzymes required for aromatic amino acid [1] and nitric oxide [2] metabolism. Although BH4 was originally discovered more than a hundred years ago [3] as a pteridine [4] from the wing (Greek name “ptera”) of the English butterfly, its biological function remained unknown until the late 1950s when it was first found [5] to be a cofactor of aromatic amino acid hydroxylase enzymes. As a cofactor, BH4 binds to the non-heme iron center of aromatic amino acid hydroxylases [6] such as phenylalanine, tyrosine, and tryptophan hydroxylases, facilitates oxygen binding to the enzyme, and causes subsequent hydroxylation of amino acids to generate tyrosine and monoamine neurotransmitters such as dopamine and serotonin. By controlling the synthesis of these neurotransmitters, BH4 has emerged as an important regulator of neuromotor and neurocognitive functions of the brain.

The potential implication of BH4 in cardiovascular health was apparent in the early 1990s, when BH4 was found to upregulate nitric oxide synthesis via the stimulation of calcium/calmodulin-dependent constitutive nitric oxide synthase (NOS) enzymes [7,8]. As a cofactor, BH4 increases the spin rate of a non-heme iron in the catalytic center of the NOS enzyme, resulting in the enhancement of its catalytic activity [9]. The role of BH4 in cellular inflammation also became evident once the exogenous addition of BH4 in IFNγ-stimulated RAW macrophages was found [2] to induce the synthesis of nitrite and nitrate, leading to the discovery of its role as a cofactor of inducible nitric oxide synthase enzyme (iNOS). The activation of the iNOS enzyme is directly linked to the induction of chronic inflammation [10]. Hence, the role of BH4 metabolism cannot be underestimated in the pathogenesis of chronic inflammatory diseases.

The earliest account of BH4 dysregulation in chronic illness was reported when plasma levels of BH4 were found to be upregulated by 150% in 20 patients with chronic depression [11]. Although BH4 has many beneficial roles in health and disease, the metabolic upregulation of BH4 may disrupt the biopterin homeostasis, resulting in the induction of a myriad of chronic conditions including pain, autoimmune ulceritis, arthritis, and inflammation [12].

Since BH4 has a profound role in regulating cardiovascular health [4], we were interested in studying the metabolism of BH4 in a subset of ME/CFS patients with orthostatic intolerance (OI). OI is a chronic metabolic condition that severely impairs cardiovascular health in ME/CFS patients.

While Myalgic Encephalomyelitis/Chronic Fatigue Syndrome (ME/CFS) is considered a biological condition, its precise cause is uncertain. Identifying a single etiology for ME/CFS is challenging due to its complicated pathophysiology which involves numerous interrelated systems. Increased inflammation is one of the characteristics that ME/CFS has in common with autoimmune illnesses [13]. Studies have revealed increased cytokine levels together with anomalies in T-cells and natural killer cells. Immune system dysfunction brought on by infections may result in chemical alterations in the brain that produce ME/CFS symptoms [14]. There is evidence linking ME/CFS to mitochondrial malfunction, which can result in ineffective respiration and compromised TCA cycle substrate supplies [13]. Ion abnormalities in skeletal muscles brought on by ME/CFS may result in calcium overload, vascular and mitochondrial dysfunction, and insulin resistance in the muscles [15]. According to research that used functional magnetic resonance imaging (fMRI) brain scans, individuals with ME/CFS exhibit less activity in the brain’s temporal-parietal junction (TPJ) [14]. This could cause weariness by interfering with the brain’s ability to determine how much effort it has to put out. Metabolic problems, aberrant reactions to oxidative stress, and hormonal abnormalities are other factors that may contribute to the development of ME/CFS. In addition to being brought on by a viral or other infection [16], ME/CFS symptoms can also be brought on by surgery, physical stress, or a shift in hormone levels [17].

Since ME/CFS cannot be definitively diagnosed by a single lab test or biomarker, unlike many other conditions, the diagnosis is dependent mostly on clinical presentation and symptom history, which can be subjective and interpretive [18]. It can be challenging to distinguish ME/CFS from other illnesses because many of its symptoms, such as exhaustion, muscle soreness, cognitive impairment, cardiovascular alterations, and sleep abnormalities, are also present in other prevalent conditions [19]. It is difficult to develop uniform diagnostic standards for ME/CFS because the severity and presentation of symptoms might differ greatly amongst patients. Additionally, there may be a lack of knowledge among healthcare workers: misdiagnosis, dismissing symptoms, and delays in receiving the right care might result from certain medical personnel’s inadequate knowledge of ME/CFS [20]. Due to the heterogeneous nature of disease symptoms, diagnosis, and etiologies, understanding the molecular mechanisms of ME/CFS is a complicated process.

Interestingly, our studies [21,22] revealed that ME+OI patients displayed a strong elevation of BH4 and its downstream derivative dihydrobiopterin (BH2) in their serum samples, suggesting that the potential metabolic dysregulation of biopterin biosynthesis could play a critical role in ME+OI pathogenesis. Moreover, when these serum samples were exogenously added to human microglia, we observed a strong correlation between elevated biopterins and the oxidative stress response, confirming a direct link between the metabolic dysregulation of BH4 and the oxidative stress response in OI patients. While exploring the molecular mechanism of BH4 upregulation, our recent study [21] demonstrates that the induction of the anaerobic pentose phosphate pathway (PPP), an alternative pathway of glucose metabolism, could be the upstream regulator for the elevated levels of pteridine metabolites in OI patients. By introducing a novel cell culture strategy, we have demonstrated how the implementation of a less-oxygenated environment followed by the induction of the non-oxidative PPP redirects the utilization of ribose-5-phosphate to the formation of BH4 via the purine biosynthetic pathway.

Detection of biopterin metabolites in plasma samples is complicated due to the labile nature of biopterin intermediates and heterogeneous sample collection procedure. Therefore, in our current perspective article, we first demonstrate the feasibility and combinations of different strategies to quantify biopterins in ME/CFS plasma and then discuss how BH4 metabolism plays a critical role in the pathogenesis of ME/CFS.

## 2. BH4 Biosynthesis in a Nutshell

**Figure 1:**
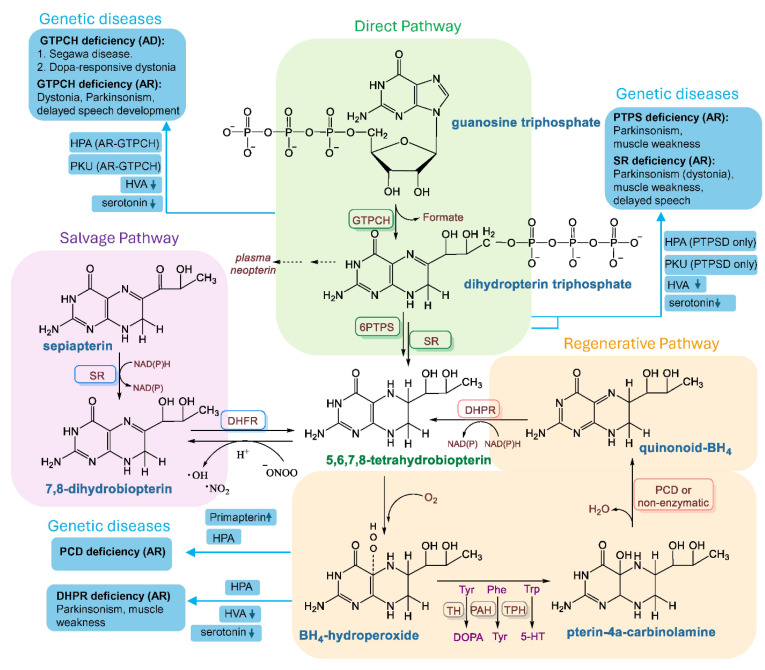
Biosynthesis of BH4 and genetic diseases due to BH4 deficiency. (Green enclosure) De novo or direct pathway of BH4 biosynthesis. Guanosine triphosphate (GTP) is enzymatically converted to BH4 by the successive enzymic actions of GTP cyclohydrolase (GTPCH1), 6-pyruvoyl-tetrahydrobiopterin synthase (6PTPS or PTPS), and sepiapterin reductase (SR). Autosomal dominant (AD) and autosomal recessive (AR) mutations of the gtpch1 gene cause BH4 deficiency via the de novo biogenesis pathway. PTPS deficiency and SR mutations are two other AR traits for BH4 deficiency. HPA = hyperphenylalaninemia, PKU = phenylketonuria, HVA = homovanillic acid. (Purple enclosure) Salvage pathway of BH4 biosynthesis. In this metabolic pathway, sepiapterin is first converted to 7,8-dihydrobiopterin (BH2) by sepiapterin reductase (SR) and then BH2 is converted to BH4 by dihydrofolate reductase (DHFR). BH4 is non-enzymically converted to BH2, contributing to the generation of reactive oxygen species. (Orange enclosure) Regenerative pathway. In the regenerative pathway, BH4 is regenerated in two reactions. BH4 is first oxidized to BH4-hydroperoxide and then converted to pterin-4a-carbinolamine during catalysis by aromatic amino acid hydroxylases such as tyrosine, phenylalanine, and tryptophan hydroxylases. Pterin-4a-carbinolamine is then converted to q-BH2 (Quinonoid BH2) by pterin-4a-carbinol- amine dehydratase (PCD) and then re-cycled back to BH4 by dihydropteridine reductase (DHPR). PCD deficiency (AR) causes the upregulation of the pterin metabolite primapterin and HPA. DHPR deficiency (AR) causes the loss of HVA, serotonin, and HPA. The flowchart of the BH4 metabolism is illustrated as described in Chapter 6 of the book “*Nitric Oxide (2nd edition)*” [23].

The biosynthesis of tetrahydrobiopterin (BH4) is a complex metabolic process that combines three different pathways, based on the metabolic demands of a cell. These are the direct or de novo, salvage, and regenerative pathways. Under physiological conditions, de novo biosynthesis is operative, in which a purine metabolite GTP is converted to BH4 by the action of three different enzymes, including a rate-limiting enzyme called GTP cyclohydrolase1 (GCH1 or GTPCH1), 6-Pyruvoyltetrahydropterin synthase (6PTPS or PTPS), and sepiapterin reductase (SR) (Figure 1; *green enclosure*). However, during regenerative and restorative conditions, when a cell recovers from stress and inflammation, both the regenerative and salvage pathways start functioning. In the salvage pathway, BH4 is regenerated from dihydrobiopterin (BH2) by the enzymic action of dihydrofolate reductase (DHFR) (Figure 1; *purple enclosure*), whereas in the regenerative pathway, quinonoid-BH4 is converted to BH4 by the action of dihydropteridine reductase (DHPR) (Figure 1; *orange enclosure*). Apart from these three pathways, BH4 biosynthesis is tightly regulated by glucose, amino acid, and nucleotide metabolisms. Metabolic pathways, including glycolysis, the pentose phosphate pathway, folic acid metabolism, and the biosynthesis of purine nucleotides indirectly but critically control the biogenesis of BH4.

## 3. Deficiency of BH4 in Disease

As a cofactor, BH4 catalyzes the enzymic activities of all hydroxylase enzymes required for the downstream conversion of aromatic amino acids, such as phenylalanine, tyrosine, and tryptophan, to their respective amines, such as tyrosine, dopamine, and serotonin. BH4 also regulates the production of the second messenger nitric oxide (NO) by the enzymic conversion of L-arginine to citrulline. Therefore, BH4 deficiency directly suppresses the synthesis of monoamine neurotransmitters, causing broad-spectrum neurological abnormalities, builds up the phenylalanine in the body causing phenylketonuria, and reduces citrulline, causing hypocitrullinemia in mitochondrial encephalopathy, lactic acidosis, and stroke-like episodes [24]. In addition to all these clinical manifestations, BH4 deficiency also causes the vascular phenotype of hypertension. Reduced NO production due to BH4-eNOS uncoupling impairs smooth muscle relaxation in the endothelium of blood vessels, causing high blood pressure or hypertension.

BH4 deficiency is often reported due to genetic mutations in BH4 biosynthetic genes. According to consensus guidelines [25], there are five autosomal recessive (AR) forms and one autosomal dominant (AD) form of genetic disorders causing BH4 deficiencies. Key enzymes, including GTP cyclohydrolase 1 (encoded by *gtpch1* gene; AD or AR), 6-pyruvoyl tetrahydropterin synthase (encoded by *ptps* gene; AR), sepiapterin reductase (encoded by *sr* gene; AR), quinoid-dihydropteridine reductase (*dhpr* gene; AR), and pterin-4-alpha carbinolamine dehydratase (*pcd1* gene; AR), are reported to be mutated, causing BH4 deficits (Figure 1). Except autosomal dominant *gtpch1* (AD-GTPCH1) and autosomal recessive *sr* mutations, hyperphenylalaninemia (HPA) or the overexpression of phenylalanine is the most common biochemical change in all of these genetic disorders of BH4 deficiency. Downregulations of monoamines such as serotonin and homovalinic acid are also frequently reported. Clinically, these genetic alterations display mostly Parkinson’s disease-like motor impairments such as dystonia, ataxia, gait difficulties, dyskinesia, and other involuntary movements along with broad-spectrum neurological abnormalities, including impaired speech development, cognitive impairment, and epilepsy. Hypersalivation, swallowing difficulties, and the loss of muscle tone are also frequently observed.

However, apart from these genetic mutations, the metabolic depletion of BH4 is possible due to its increased oxidation to dihydrobiopterin (BH2), which may contribute to the oxidative stress in ischemic injury [26], neurodegeneration [27], inflammation [28], and cardiovascular disease [29,30]. Although the molecular mechanism is not yet established, mitochondrial degeneration due to stress, inflammation, and injury could be an upstream trigger. The enhanced electron leakage [31] due to the mitochondrial impairment of energy metabolism and the subsequent generation of reactive oxygen species (ROS) potentially stimulates the conversion of BH4 to BH2. The conversion is a non-enzymic process and potentially generates reactive free radicals.

## 4. Detection of BH4: A Practical Challenge in ME/CFS Plasma Samples

Because of the overwhelming evidence of BH4 deficiency in cardiovascular and metabolic diseases, we were interested in measuring BH4 levels in ME/CFS patients by different techniques. To minimize the heterogeneity of the sample collection procedure, all plasma samples were collected at the same time, from the same geographical location, after matching ethnicity, age, and genders between controls and cases. We first conducted a comprehensive ELISA analysis to measure the level of BH4 in freshly harvested plasma samples of ME/CFS subjects and age-/gender-matched controls. Surprisingly, our pilot ELISA analyses revealed that the BH4 level was not impaired but highly elevated in ME+OI subjects compared to the age- and gender-matched controls [14,21]. The raw data of ELISA analyses and the step-by-step strategy of scale conversion from μg/mL to pmole/mg unit were made available in a Mendeley open data source file (https://data.mendeley.com/datasets/4jjngjrn86/1, the content created on 21 July 2021 and deposited on 7 November 2024). The pmole/mg unit is a widely accepted unit for measuring biopterins, as reported in other studies [32,33,34,35], and our detected levels fall within that reported range. Based on our pilot study, the expression of downstream BH2 was also found to be elevated, suggesting that the dysregulated biopterin metabolism could be a very critical contributing factor in ME/CFS pathogenesis. ELISA detection of small molecules can be erroneous, so we repeated the standard curve with commercially available BH4 and BH2 and then confirmed our results. However, the finding was unexpected and encouraged us to adopt a different technique for the detection of both BH4 and BH2.

**Figure 2:**
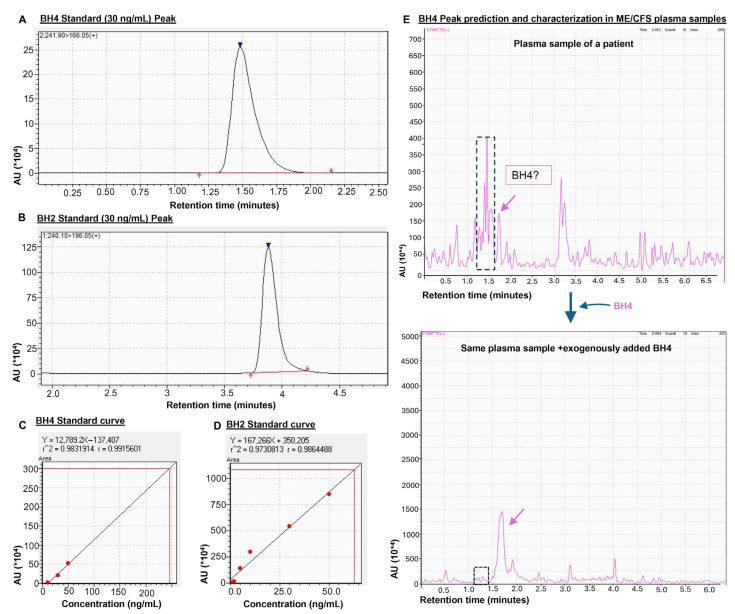
The potential challenge of detecting BH4 in plasma samples by a triple-quad LCMS-8040 strategy. (**A**) Representative peaks for purified BH4 (30 ng/mL; Cat# T4425-5MG; Millipore Sigma Aldrich, St. Louis, MO, USA) and (**B**) BH2 (30 ng/mL; Cat# 37272-10MG; Millipore Sigma Aldrich) dissolved in 0.002% formic acid in D.I. water. Ionization energy method = electrospray ionization; column type = Gemini 5 µm C18 110 Å LC Column 150 × 2 mm (Cat# 00F-4435-B0, Phenomenex, Torrance, MD, USA); mobile phase composition = binary solvent system, solvent A: 0.002% formic acid in water; solvent B: 0.002% formic acid in methanol. Standard curves for (**C**) BH4 and (**D**) BH2. (**E**) The electrospray ionization (nebulizing gas flow 2 L/min and drying gas flow 15 L/min) and heat (DL temperature 250 °C and heat block temperature 400 °C) generated during the run destabilized BH4. Therefore, it was extremely difficult to identify the BH4 peak. The demonstrated peak (pink arrow; upper spectrogram) is too small to be considered as a real peak. However, the exogenous addition of BH4 increased the peak height (pink arrow; lower spectrogram) without altering the adjacent peaks (enclosed in a dotted rectangle), suggesting that the peak could be a BH4 peak. In chromatograms, AU = arbitrary unit and “*10^4^” of AU indicated 10,000 times of the displayed unit.

Liquid chromatography mass spectrometry (LCMS) is a technique routinely used in various applications of analytical chemistry and is so far considered as a gold-standard technique for the measurement of small molecular compounds such as pterin metabolites in plasma samples. Therefore, next we conducted LCMS analysis in plasma samples to detect biopterins. The sample preparation, optimization of column parameters, loading to the column, detection, and peak identification were performed using a protocol written elsewhere [36]. Briefly, the freshly harvested blood samples were processed for plasma in a BD Vacutainer® Plasma Preparation Tube (BD Biosciences, Franklin Lakes, NJ, USA) (clotting time 30 min) and the separated plasma was immediately processed. Hydrochloric acid (0.1 M) was added to precipitate protein, the sample was centrifuged at 12,000 rpm for 10 min, and the supernatant was collected, dried up by passing nitrogen gas at a flowrate of 1.5 L/min at r.t., reconstituted with 0.002 (*v*/*v* in H_2_O) formic acid (mobile phase), passed through a spinX filter, and then loaded in a HPLC column. The iodine solution recommended in the original protocol [22] was not added to avoid oxidation. The running parameters are mentioned in the legend of Figure 2.

However, this technique has its own limitations as well. The electron ionization of a molecule often leads to a diverse range of chemical modifications, including dehydrogenation, dehydration, oxidation, and reduction. Therefore, it is extremely hard to identify a chemically unstable or labile molecule like BH4 by this technique. Before analyzing samples, we performed standard curve analyses (Figure 2A–D) to optimize, identify, and quantify BH4 (Figure 2A,C) and BH2 (Figure 2B,D). When BH4 was loaded to the column, the electrospray ionization rapidly destabilized the BH4, which produced two identifiable peaks, one near 1.5 min characteristic of BH4 and another around 3.9 min characteristic of BH2. The dose curve analysis further revealed that BH4 can be detected at concentrations as low as 10 nM with our optimized protocol. However, if the electrospray ionization MS (ESIMS) analysis of a plasma sample displays a peak with a height lower than the minimum detectable dose, it is extremely hard to confirm if that peak is truly for BH4. One strategy to circumvent this potential issue is to add BH4 exogenously to the sample and then analyze by LCMS. If the addition of BH4 increases the height of that particular peak without altering other peaks, then the identified peak is considered as the potential peak for BH4. Interestingly, when analyzing plasma samples in LCMS, we identified a small peak (Figure 2E; top) near 1.5 min for plasma samples of ME+OI patients. Adding BH4 exogenously increased the height of that small peak (Figure 2E; bottom), suggesting that the peak around 1.5 min could be the peak for BH4.

**Figure 3:**
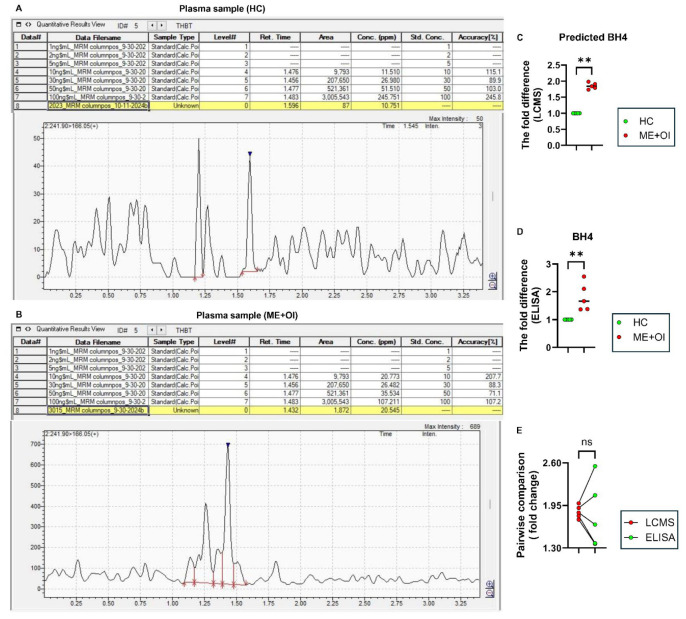
The comparative analysis of BH4 by LCMS and ELISA. Freshly harvested plasma samples were analyzed for LCMS and ELISA to compare the relative levels of BH4 (blue arrowheads) between healthy control (HC) and ME/CFS with orthostatic intolerance (ME+OI) groups (*n* = 5 per group). Representative mass spectrograms are displayed after analyzing plasma samples of (**A**) HC and (**B**) age-/gender-matched ME+OI subjects. The fold difference of BH4 levels was analyzed between the HC and age-/gender-matched ME+OI cases by (**C**) LCMS and (**D**) ELISA. The Mann–Whitney U test demonstrates the significance of the mean between the two groups with ** *p* < 0.01 = 0.0079. (**E**) Pairwise analyses of the same samples between LCMS and ELISA displays a similar trend (ns = no significance). In each table, the numbers under “Area” column are annotated by comma (,) for every 1000 unit. All other numbers under Retention time, concentrations, and accuracy columns are represented in decimal unit.

**Figure 4:**
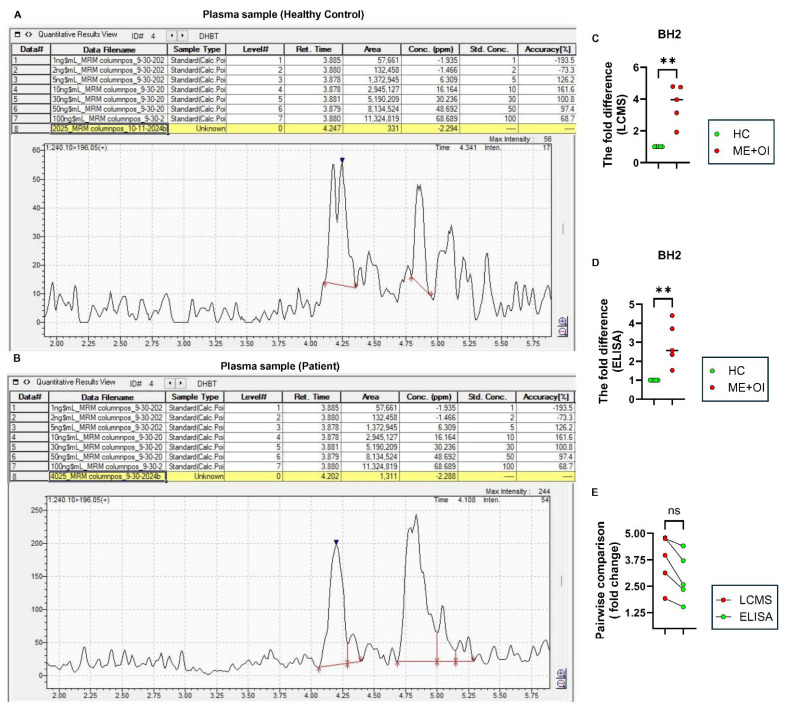
The comparative analysis of BH2 by LCMS and ELISA. Freshly harvested plasma samples were analyzed for LCMS and ELISA to compare the relative levels of BH4 (blue arrowheads) between healthy control (HC) and ME/CFS with orthostatic intolerance (ME+OI) groups (*n* = 5 per group). Representative mass spectrograms are displayed after analyzing plasma samples of (**A**) HC and (**B**) age-/gender-matched ME+OI subjects. The negative value in concentration (ppm) means that the BH2 peak area of the sample is below the chosen range of standard concentrations. It does not mean that BH2 was undetected. In these cases, area is considered. The fold difference of BH2 levels was analyzed between the HC and age-/gender-matched ME+OI cases by (**C**) LCMS and (**D**) ELISA. The Mann-Whitney U test demonstrates the significance of the mean between the two groups with ** *p* < 0.01 = 0.0079. (**E**) Pairwise analyses of the same samples between LCMS and ELISA displays a similar trend (ns = no significance). In each table, the numbers under “Area” column are annotated by comma (,) for every 1000 unit. All other numbers under Retention time, concentrations, and accuracy columns are represented in decimal unit.

Subsequent LCMS analyses of BH4 in *n* = 5 HC and *n* = 5 age-/gender-matched ME+OI patients followed by peak integration statistics supported our previous finding of significantly elevated BH4 (Figure 3) and BH2 (Figure 4) levels in ME+OI plasma samples compared to HC plasma samples. Interestingly, when measuring the fold-change difference between the HC and ME+OI cases, both LCMS (Figure 3C) and ELISA (Figure 3D) displayed similar differences (an approximately 2-fold change). The pairwise comparison (Figure 3E) of BH4 levels detected in LCMS and ELISA for the same samples further corroborated the consistency between two techniques. Similarly, LCMS (Figure 4A–C), ELISA (Figure 4D), and a pairwise comparison between the two techniques (Figure 4E) confirmed that ME+OI plasma samples had significantly higher BH2 levels.

Both ELISA and LCMS have their own limitations measuring pterin metabolites in terms of accuracy and sensitivity. Therefore, combining results from both analyses, we concluded that biopterin metabolism may be dysregulated in ME+OI cases.

## 5. Regulation of BH4 Biosynthesis in ME/CFS

Before diving deep into the molecular mechanism of BH4 biosynthesis in ME/CFS pathogenesis, we must review some published literature relevant to our study.

Until now, a significant amount of research has been performed to investigate the role of energy metabolism in ME/CFS pathogenesis. An NMR-based comprehensive metabolomic study [37] demonstrated that ME/CFS patients might be associated with reduced energy production via the impairment of glycolysis. Fluge et.al. [38] reported that the pyruvate dehydrogenase (PDH) enzyme complex was impaired in ME/CFS patients. As a result, the glycolytic end-product pyruvate could not be converted to acetyl CoA for subsequent energy production in mitochondria. Nevertheless, another study [39], with the help of seahorse respirometry, demonstrated that the reduced mitochondrial consumption of oxygen led to a deficit of energy production. While glucose metabolism via glycolysis was found to be downregulated, a genomic study demonstrated that genes of the pentose phosphate pathway (PPP) were upregulated in ME/CFS patients [40]. Interestingly, our cDNA-based array analysis in PAXgene-separated RNA samples followed by enzyme activity assay in PBMCs demonstrated that several genes and proteins of the PPP such as glucose-6-phosphate dehydrogenase (G6PDH), transaldolase (TALDO), and transketolase (TK) were upregulated and activated during ME/CFS pathogenesis [21]. This initial finding intrigued us to study the biochemistry of the PPP in detail.

**Figure 5:**
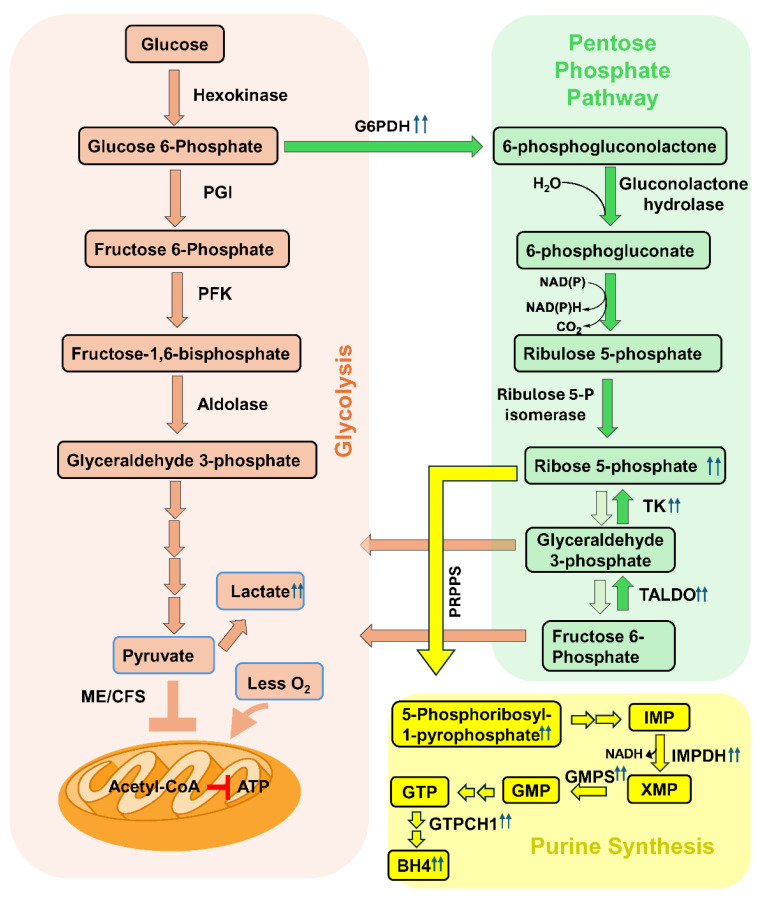
Integrated metabolic pathways of BH4 metabolism in ME/CFS pathogenesis. (*Red enclosure*) The glycolysis pathway of glucose metabolism leads to the formation of pyruvate. Pyruvate is transported to mitochondria, converted to acetyl CoA, and metabolized by the TCA cycle, generating reduced intermediates for ATP production. The utilization of glucose by glycolysis is compromised in ME/CFS pathogenesis. Reduced oxygen consumption by mitochondria was reported to uncouple the electron transport chain for ATP synthesis. (*Green enclosure*) Augmented pentose phosphate pathway by the enzymic activation of glucose-6-phosphate dehydrogenase (G6PDH), Transketolase (TK), and transaldolase (TALDO). Reduced oxygen consumption by mitochondria and enhanced bioavailability of NADPH may induce the reduction potential in the cell, causing reversible enzymes TALDO and TK to increase the biogenesis of ribose-5-phosphate (R5P). (*Yellow enclosure*) Synthesis of purine metabolites. Phosphoribosyl pyrophosphate synthase (PRPPS) enzyme converts R5P to inosine-5-monophosphate (IMP), which is converted to GMP by the successive enzymic actions of IMP dehydrogenase (IMPDH) and GMP synthase (GMPS). GTP is synthesized by GTP, which generates BH4 via the direct pathway by the enzymic action of GTPCH1. R5P is the direct precursor for purine biosynthesis. Once the bioavailability of R5P increases and its utilization via glycolysis is inhibited, it is expected that the downstream formation of purine metabolites via the nucleotide biosynthetic pathway (*yellow enclosure*) will be operative. Phosphoribosyl pyrophosphate synthase (PRPPS) is the first enzyme of the purine biosynthetic pathway that catalyzes the synthesis of 5-phosphoribosyl-1-pyrophosphate (PRPP) from R5P. Previous studies reported that the induction of hypoxia enhanced the enzymic activity of PRPPS [52]. Accordingly, our biochemical assay also identified a very strong level of PRPP in the microglial cells when a hypoxic or less-oxygenated environment was implemented [21]. Moreover, we also demonstrated that inosine-5-monophosphate dehydrogenase (IMPDH), a rate-limiting enzyme of purine biosynthesis, and GMP synthase (GMPS), another key enzyme of purine biogenesis, were upregulated in hypoxic condition. Interestingly, inhibition of the non-oxidative PPP by *taldo1* siRNA also attenuated the expression of both IMPDH and GMPS in microglia. Collectively, these observations demonstrated that the augmentation of the non-oxidative PPP by hypoxia-induced strong reductive potential guided R5P for purine biogenesis.

The PPP starts from glycolytic intermediate glucose-6-phosphate as a precursor (Figure 5; *green shade*), runs in parallel to glycolysis (Figure 5; *red shade*), and shunts carbon back to glycolysis via generating a series of four- and five-carbon sugar intermediates. It is a compensatory and alternative strategy for glucose metabolism if glycolysis is impaired [41,42]. However, if mitochondria are deficient in oxygen consumption or the PDH enzyme complex on the mitochondrial membrane cannot efficiently convert pyruvate to acetyl CoA, then the activation of the PPP could also be ineffective for downstream energy production.

The PPP can operate in two different phases, including the oxidative or aerobic phase and the non-oxidative or non-aerobic phase. The oxidative PPP is the first phase of the PPP and efficiently shunts carbon to glycolysis via the production of ribulose-5-phosphate and NADPH. It is oxidative because glucose-6-phosphate is oxidized to produce lactone by generating one molecule of reduced NADPH. On the other hand, the non-oxidative or non-aerobic PPP stage operates in the second phase of the PPP and generates a series of C5 and C4 sugar intermediates to finally meet glycolysis for energy production. There are two critical enzymes, known as transaldolase (TALDO) and transketolase (TK), that reversibly guide the synthesis and turnover of ribose-5-phosphate (R5P) in the non-oxidative phase of the PPP.

The augmented bioavailability of R5P facilitates the synthesis of purine nucleotides and eventually the biogenesis of BH4 (Figure 5; *yellow shade*). In fact, our observation [21] regarding the potential upregulation of purine biosynthesis and metabolic biproducts of purine metabolism was further corroborated in ME/CFS plasma samples by a high-resolution metabolomic study [43]. To further investigate the pathogenic role of upregulated purine in ME/CFS, we were interested to introduce a metabolic condition that upregulates the bioavailability of R5P. The enzymic actions of both TALDO and TK are redox-sensitive [44,45] and guided by mitochondrial membrane potential [46]. Studies have shown that the implementation of a less-oxygenated or hypoxic environment induces the enzymic activity of TK [47] and TALDO [48] in cancer cells. In agreement with this observation, we devised a novel cell culture model in which human microglial cells were treated with exogenous R5P and then kept in a hypoxic chamber supplemented with 85%N_2_/10%CO_2_/5%O_2_ for 24 hrs. This strategy implemented a strong reductive potential in the cell that not only activated the enzymic activities of TALDO and TK, but also essentially biased the activity of TALDO and TK to one direction, leading to the synthesis of endogenous R5P from C4 and C5 intermediates (Figure 5; *bottom half of green enclosure*). Therefore, by implementing the hypoxic environment and supplementing R5P exogenously, we were able to upregulate the bioavailability of R5P. Moreover, the enzymic activity of G6PDH, the rate-limiting enzyme of the PPP, was also found to be enhanced in our novel cell culture model. Interestingly, upregulations of G6PDH, TALDO, and TK were found to be involved in ME+OI pathogenesis [21]. Therefore, we considered that our cell culture model is pathologically relevant to ME/CFS. In addition to that, the implementation of hypoxic environment impairs oxidative metabolism in mitochondria [49] (Figure 5; *red enclosure, bottom part*), which is frequently reported in ME/CFS pathogenesis [39,50,51].

GTP, a purine metabolite, is converted to BH4 via the direct or de novo pathway by the enzymic action of GTPCH1 (Figure 1; *green shade*). We observed that the induction of the non-oxidative PPP followed by the addition of R5P also upregulated the synthesis of GTPCH1 [21] and the knocking down of the *taldo1* gene significantly ameliorated the expression of GTPCH1 and subsequent production of BH4. Therefore, our research suggests that the induction of reductive potential upregulated the BH4 biosynthesis via the augmentation of the anaerobic PPP followed by purine biosynthesis pathways. Interestingly, our PAXgene RNA array followed by ELISA assay also demonstrated that BH4 biosynthetic genes such as *dhfr* and *dhpr* were found to be strongly upregulated in ME+OI subjects. *Dhfr* gene encodes for the dihydrofolate reductase (DHFR) enzyme, which converts 7,8-dihydrobiopterin (BH2) to BH4 via the salvage pathway (Figure 1; purple enclosure), whereas the dhpr gene encodes for dihydropteridine reductase (DHPR), which regenerates BH4 from quinonoid BH2 (Figure 1; *orange enclosure*).

Collectively, our recent studies demonstrate strong evidence for the upregulation of BH4 in ME+OI patients.

## 6. The Potential Role of Biopterins (BH4 and BH2) in the Pathogenesis of ME/CFS

ME/CFS is a chronic multi-system illness that compromises the quality of a healthy life by causing long-term muscle fatigue, pain, dizziness, and confusion. Clinically, the illness is diagnosed by post-exertional fatigue and orthostatic intolerance manifested by a sudden drop [53] in blood pressure while maintaining an upright posture. Recent studies [53,54,55] indicate that the reduction of effective blood flow in the cranial blood vessel could manifest an unexpected drop in blood pressure, causing dizziness and fainting. While the molecular mechanism of the reduced blood pressure is still unknown, the dilation of blood vessels or vasodilation could be one such mechanism that causes a decrease in systemic vascular resistance resulting in the reduction of blood pressure. The role of BH4 in vasodilation has been well-documented [56,57,58] since its discovery as a cofactor of endothelial nitric oxide synthase (eNOS or NOS3) enzyme. The activation of eNOS by BH4 followed by the upregulated catalytic conversion of L-arginine to nitric oxide directly causes the dilation of smooth muscle in blood vessels via the soluble guanylate cyclase-cGMP-PKG pathway [59,60], resulting in the reduction of blood pressure. The induction of this cellular event is a beneficial strategy to alleviate vascular hypertension. However, uncontrolled activation of BH4 biosynthesis could display an adverse effect of chronic hypotension. Moreover, BH4 also catalyzes the production of NO via the activation of inducible nitric oxide synthase (iNOS or NOS2) enzyme in macrophages [61], microglia [62], and dendritic cells [63]. INOS-derived NO plays a direct role in inducing stress and inflammation via the generation of reactive nitrogen (RNS) and oxygen species (ROS) [64,65,66]. Therefore, metabolic dysregulation resulting in the elevated expression of BH4 not only manifests orthostatic hypotension but could also augment nitrosative stress and inflammation.

**Figure 6:**
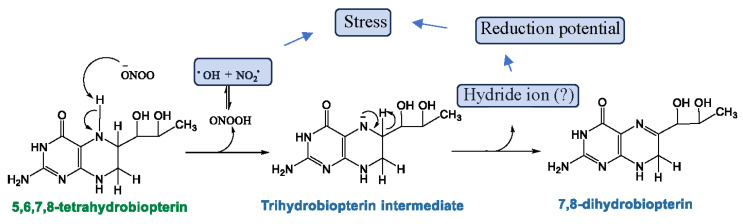
Non-enzymic conversion of BH4 to BH2. A schema that displays a potential metabolic conversion of BH4 to BH2. Nucleophilic attack by peroxynitrite (OONO^−^) at the N5 hydrogen of BH4 may generate a trihydrobiopterin intermediate, releasing OH and NO_2_ radicals. BH3 quickly converts to BH2, potentially by generating a hydride ion and further increasing the reduction potential in the cell.

**Figure 7:**
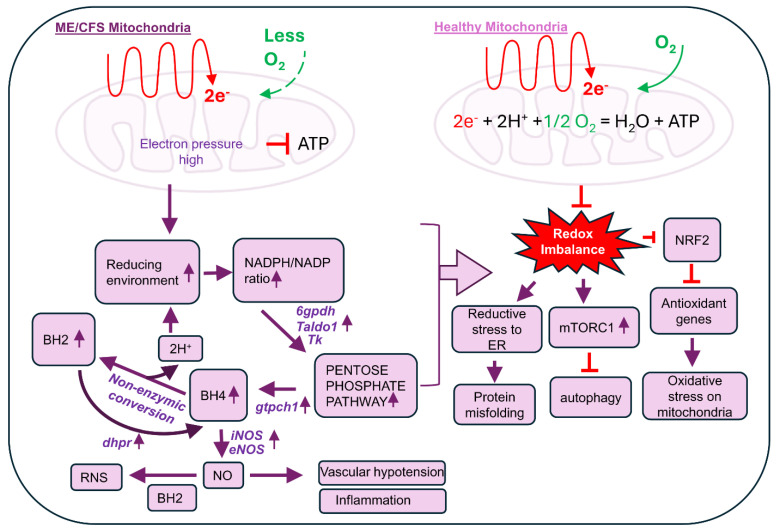
The metabolic contributions of elevated biopterins in the pathogenesis of ME/CFS. Reduced mitochondria consumption of oxygen followed by the impairment of the electron transport chain increases electron pressure in mitochondria, affects the redox balance in cells, causing the augmentation of the non-oxidative pentose phosphate pathway, and may lead to the dysregulation of biopterin homeostasis. The potential redox imbalance increases reactive stress in the endoplasmic reticulum and disrupts the glutathione homeostasis, causing protein misfolding. Reactive stress also activates mTORC1, leading to autophagy impairment. Cellular increase in reductive potential causes the inactivation of NRF2 and downregulation of anti-oxidant genes in mitochondria. All these phenomena may lead to the augmentation of stress and inflammation.

For the induction of nitrosative stress, the metabolic balance between BH4 and BH2 is critical (Figure 6). According to our recent findings, we predict that the enhanced biogenesis of BH4 upregulates the production of NO, which is followed by the formation of reactive nitrogen species such as peroxynitrite (OONO^−^) due to impaired mitochondrial energy metabolism. These reactive nitrogen metabolites facilitate the non-enzymic conversion of BH4 to BH2 by generating the unstable intermediate BH3 (Figure 6). The non-enzymic conversion of BH4 to BH2 not only generates more reactive free radicals, but also potentially adds to the reduction potential in cell by generating protons (H^+^). Increased protons contribute to the induction of reductive stress in cell. In fact, the augmented PPP followed by the generation of reduced species (H^+^ and NADPH) has been previously shown [67] to induce reductive stress in cell. Our cDNA-based array analysis of biopterin synthetic genes followed by a biochemical analysis of biopterin metabolites revealed that the metabolic interconversion of BH4 and BH2 was highly operative in ME/CFS pathogenesis. Upregulated BH4 biosynthesis led to the non-enzymic conversion of BH2 and BH2 reformed BH4 via the enzymic upregulation of DHPR. This cyclic process potentially induces metabolic disturbances via inducing reductive stress in various mechanisms (Figure 7). *First*, the increased reduction potential could dysregulate glutathione metabolism and generate reductive stress in the endoplasmic reticulum (ER), causing protein misfolding. *Second*, the induced reductive stress is known to activate the mammalian target of the rapamycin 1 (mTORC1) complex [68], causing autophagy impairment [69]. Our previous study [70]) also demonstrated that ME/CFS pathogenesis is associated with increased mTORC1 activation followed by autophagy impairment. *Finally*, as demonstrated previously [71] in other metabolic diseases, increased reductive stress can inhibit mitochondrial protein NRF2 and suppress expressions of the NRF2-dependent anti-oxidant gene, contributing directly to the oxidative stress response in ME/CFS pathogenesis.

## 7. Summary

The metabolism of BH4 is tightly regulated in health and disease. While BH4 deficiency is mostly genetic, chronic metabolic diseases such as cancer and inflammation sometimes cause the depletion of BH4 due to its oxidative conversion to other biopterin metabolites. However, in ME/CFS, neither deficiency nor depletion was observed. Instead, we observed that the biosynthesis of BH4 and its turnover to BH2 maintained a cyclic metabolic process that perpetuates oxidative stress and the inflammatory response in ME/CFS patients. While shedding light on biopterin biosynthesis, our research identified that anaerobic glucose metabolism could be an upstream pathway. The upstream impairment of oxidative glucose metabolism and mitochondrial deficit of energy production followed by the induction of a reducing environment could be responsible for the dysregulation of BH4 metabolism. Taken together, our current review article unfurls the molecular regulation of BH4 metabolism in the induction of stress, inflammation, and cardiovascular pathologies in ME/CFS.

## Data Availability

There is no electronic datasheet associated with this paper. No data are in any electronic repository.
